# High Resolution Turntable Radar Imaging via Two Dimensional Deconvolution with Matrix Completion

**DOI:** 10.3390/s17030542

**Published:** 2017-03-08

**Authors:** Xinfei Lu, Jie Xia, Zhiping Yin, Weidong Chen

**Affiliations:** 1 Key Laboratory of Electromagnetic Space Information, Chinese Academy of Sciences, University of Science and Technology of China, Hefei 230027, China; lxfei@mail.ustc.edu.cn (X.L.); jiexia@mail.ustc.edu.cn (J.X.); 2 Academy of Photoelectric Technology, Hefei University of Technology, Hefei 230009, China; zpyin@hfut.edu.cn

**Keywords:** radar imaging, deconvolution, matrix completion, undersampled data

## Abstract

Resolution is the bottleneck for the application of radar imaging, which is limited by the bandwidth for the range dimension and synthetic aperture for the cross-range dimension. The demand for high azimuth resolution inevitably results in a large amount of cross-range samplings, which always need a large number of transmit-receive channels or a long observation time. Compressive sensing (CS)-based methods could be used to reduce the samples, but suffer from the difficulty of designing the measurement matrix, and they are not robust enough in practical application. In this paper, based on the two-dimensional (2D) convolution model of the echo after matched filter (MF), we propose a novel 2D deconvolution algorithm for turntable radar to improve the radar imaging resolution. Additionally, in order to reduce the cross-range samples, we introduce a new matrix completion (MC) algorithm based on the hyperbolic tangent constraint to improve the performance of MC with undersampled data. Besides, we present a new way of echo matrix reconstruction for the situation that only partial cross-range data are observed and some columns of the echo matrix are missing. The new matrix has a better low rank property and needs just one operation of MC for all of the missing elements compared to the existing ways. Numerical simulations and experiments are carried out to demonstrate the effectiveness of the proposed method.

## 1. Introduction

Inverse synthetic aperture radar (ISAR) is an important imaging mode of radar application for detecting and recognizing moving targets. In conventional ISAR imaging, after removing the radial motion via motion compensation methods, the ISAR imaging is the same as turntable radar imaging, which uses the rotational motion to provide high cross-range resolution and a wide signal bandwidth to get high range resolution [1,2,3,4,5]. Traditional imaging methods based on the matched filter (MF) are robust, but fail to achieve good performance due to the convolution effect of target scattering coefficients and the point spread function (PSF). Additionally, one-dimensional (1D) deconvolution algorithms, such as Wiener filtering, iterative constraint deconvolution (CID), Bayesian angular superresolution algorithm, and so on, have been used in forward looking scanning radar imaging for removing the convolution effect and achieving high cross-range resolution [6,7]. Nevertheless, the deconvolution methods always need many measurements in the range frequency and cross-range time domains to ensure the performance of deconvolution. However, the resulting high sampling rate poses difficulties for raw data transmission and storage. The recently-developed compressive sensing (CS) framework can reduce the measurements, but under the situation that the target is constructed by some sparsely-distributed scattering points, and the number of scattering points is much less than the number of imaging grids [8,9]. Additionally, the CS-based algorithms need to design a very accurate measurement matrix, and their recovery quality may be seriously affected by the accuracy of the measurement matrix, which is always influenced by system errors and off-grid error [10,11,12]. Besides, the complexity of CS-based algorithms is huge, and the required signal to noise ratio (SNR) is relatively high, which results in instability in practical application.

Recently, matrix completion (MC) has been used for recovering a low rank matrix from a small set of corrupted entries by minimizing an objective function with a penalty term based on the matrix rank [13,14,15], and it has been introduced to radar applications for reducing the measurements and recovering missing data [16,17,18]. Bi et al. proposed a new high-resolution change imaging scheme based on MC and Bayesian compressive sensing for undersampled stepped-frequency-radar data [16]. Yang et al. developed the link between MC and undersampled SAR imaging and further provided a practical way to recover the data [17]. For the application of radar data completion, the strategy of random rows or columns of missing data are more often used. However, the MC will not be useful for this situation because it can not recover a row or column without any information of this row or column [14]. Therefore, [16,17] proposed the matrix reconstruction method for the echo of every row or column, and the reconstructed matrix satisfies the condition of MC because the observed data are randomly distributed in the reconstructed matrix. Both of their constructed matrices have a small size, and the low rank property may not hold. Then, Hu et al. proposed a new way of reshaping the sparse stepped frequency echo into a large-sized Hankel matrix form for ISAR imaging and improved the low rank property of the echo matrix [18]. However, both the ways of matrix reconstruction must be repeated many times in order to complete the missing rows or columns, which would greatly increase the computational burden.

Inspired by the high resolution radar imaging with 1D deconvolution operation for forward looking radar, we generalize it to 2D turntable radar imaging to achieve 2D high resolution radar imaging. Firstly, we derive the 2D convolution model for the turntable radar based on the MF algorithm and analyze the influence of azimuthal undersampled data on the deconvolution method. Then, in order to complete the missing data and improve the performance of deconvolution, we use the MC technique for missing data completion. Compared to other existing methods for real data MC, we propose an improved method for complex echo MC based on the hyperbolic tangent constraint to improve the performance of MC. In addition, we modify the way of echo matrix reconstruction to improve the low rank property of the echo matrix and need only one MC operation for all elements completion. Then, after the MC of echo, we introduce a new 2D deconvolution algorithm for improving the ill condition of deconvolution. At last, through many simulation and experimental results, we can verify the effectiveness of the proposed method.

The outline of this paper is summarized as follows. The 2D convolution signal model and direct deconvolution problem under undersampled data for turntable radar imaging are formulated in Section 2. Novel algorithms for MC and 2D deconvolution imaging are proposed in Section 3. Extensive numerical simulations and experiments are presented to verify the proposed method in Section 4. Finally, the conclusions are drawn in Section 5.

Notations used in this paper are as follows. Bold case letters are reserved for vectors and matrices, respectively. $diag\left(x\right)$ is a diagonal matrix with its diagonal entries being the entries of a vector x. $F_{2}\left\{\cdot \right\}$ and $F_{2}^{-1}\left\{\cdot \right\}$ are two-dimensional Fourier and inverse Fourier transform. $svd\left(\cdot \right)$ denotes the singular value decomposition. $\langle \cdot \rangle$, ⊙, * and ⊗ indicate the inner product, the Hadamard (element-wise) product, the convolution and the correlation operation. ${∥\cdot ∥}_{2}$, ${∥\cdot ∥}_{F}$ and ${∥\cdot ∥}_{*}$ are the $l_{2}$ norm, Frobenius norm and nuclear norm. ${\left(\cdot \right)}^{T}$, ${\left(\cdot \right)}^{H}$, ${\left(\cdot \right)}^{*}$ and Re· denote the transpose, conjugate transpose, conjugate and real part operations, respectively.

## 2. Problem Formulation

### 2.1. Turntable Radar Imaging Model

**Figure 1:**
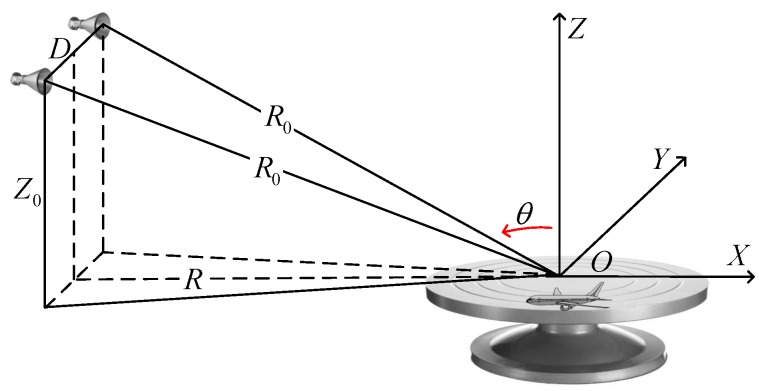
Geometry of turntable radar imaging.

Consider a typical arrangement of turntable radar given in Figure 1, which consists of the transmitting and receiving system, a high precision turntable with the target placed on it rotating with Δθ each time, respectively. A Cartesian coordinate is constructed with the center of the turntable as the origin, and the positions of the transmitting and receiving antenna are $\left(-R,\;\;-D/2\;,\;Z_{0}\right)$ and $\left(-R,\;\;D/2\;,\;\;Z_{0}\right)$, where $D,\;\;R,\;\;Z_{0}$ indicate the distance between the transmitting and receiving antennas, the distance from the antenna to the YZ and XY planes. Assuming the transmitted signal can be written as:

(1) $$s\left(t\right)=u\left(t\right)e^{j2\pi f_{c}t}$$

where $u\left(t\right)$, $f_{c}$ are the complex envelop with bandwidth B and the carrier frequency, respectively.

For the *n*-th rotating angle $\theta_{n}=n\Delta \theta ,\;\;\;\;n=1,2,\ldots ,N$, the echo reflected by the target within an imaging plane S, i.e., $\left(x,y\right)\in S$ can be represented as:

(2) $$f\left(t,\theta_{n}\right)=\underset{S}{\;\int \int \;}\sigma \left(x,y\right)u\left(t-\tau_{n}\left(x,y\right)\right)e^{j2\pi f_{c}\left(t-\tau_{n}\left(x,y\right)\right)}dxdy$$

where $\sigma \left(x,y\right)$ represents the complex reflection coefficient of the scattering point located in (x,y), and $\tau_{n}\left(x,y\right)$ is the propagation delay, which can be expressed as:

(3) $$\tau_{n}\left(x,y\right)=\frac{1}{c}\left(\begin{array}{c} \sqrt{{\left(x\cos\theta_{n}-y\sin\theta_{n}+R\right)}^{2}+{\left(x\sin\theta_{n}+y\cos\theta_{n}+D/2\right)}^{2}+Z_{0}^{2}}\\ +\sqrt{{\left(x\cos\theta_{n}-y\sin\theta_{n}+R\right)}^{2}+{\left(x\sin\theta_{n}+y\cos\theta_{n}-D/2\right)}^{2}+Z_{0}^{2}} \end{array}\right).$$

Additionally, we have the following approximation in the far-field and small rotation angle case:

(4) $$\tau_{n}\left(x,y\right)\approx \frac{2}{c}\left(R_{0}+\left(x-\theta_{n}y\right)R/R_{0}\right)$$

where $R_{0}=\sqrt{R^{2}+{\left(D/2\right)}^{2}+Z_{0}^{2}}$.

Quadrature down converted by the carrier wave $e^{j2\pi f_{c}t}$, sampling and taking the correlation operation for (1) and (2), we can get:

(5) $$z\left(t_{m},\theta_{n}\right)=\underset{S}{\;\int \int \;}\sigma \left(x,y\right)\left\{u\left(t_{m}-\tau_{n}\left(x,y\right)\right)e^{-j2\pi f_{c}\tau_{n}\left(x,y\right)}\right\}⊗u\left(t_{m}\right)\;\;dxdy.$$

The frequency spectrum of (5) can be written as:

(6) $$Z\left(f_{m},\theta_{n}\right)=\underset{S}{\;\int \int \;}\sigma \left(x,y\right)U^{*}\left(f_{m}\right)U\left(f_{m}\right)e^{-j2\pi \left(f_{m}+f_{c}\right)\tau_{n}\left(x,y\right)}dxdy$$

where $Z\left(f_{m},\theta_{n}\right)$ and $U\left(f_{m}\right)$ are the frequency spectrum form of $z\left(t_{m},\theta_{n}\right)$ and $u\left(t_{m}\right)$, $f_{m}\in \left(0,\;\;\;B\right)$ and $f_{m}=m\Delta f$, m=1,2,…,M and Δf is the frequency sampling interval.

Designing a filter function $H\left(f_{m}\right)$:

(7) $$H\left(f_{m}\right)=U^{*}\left(f_{m}\right)U\left(f_{m}\right)e^{-j2\pi \left(f_{c}+f_{m}\right)\frac{2R_{0}}{c}}$$

and applying it to (6), the frequency spectrum echo can be rewritten as:

(8) $$Y\left(m,n\right)=\underset{S}{\;\int \int \;}\sigma \left(x,y\right)e^{-j2\pi \left(f_{m}+f_{c}\right)\frac{2\left(x-\theta_{n}y\right)R/R_{0}}{c}}dxdy.$$

Under the narrow band approximation, we have $\lambda_{c}=c/\left(f_{c}+f\right)\approx c/f_{c}$, and the echo (8) can be further written as:

(9) $$Y\left(m,n\right)=\underset{S}{\;\int \int \;}e^{-j4\pi \frac{R}{R_{0}c}\left(f_{m}+f_{c}\right)x}\sigma \left(x,y\right)e^{j4\pi \frac{R}{\lambda_{c}R_{0}}\theta_{n}y}dxdy.$$

Clearly, the two dimensions of scattering data are separated for *x* and *y*, respectively. What we want to do is to recovery the 2D reflectivity $\sigma \left(x,y\right)$ from the 2D observation data *Y*.

Discretizing the continuous imaging area into P×Q grids, then (9) can be expressed in a 2D matrix form:

(10) $$Y=\Psi_{x}œ\Psi_{y}+N$$

where $\Psi_{x}\in C^{M\times P}$ and $\Psi_{y}\in C^{Q\times N}$ are the observation matrices, ${\left(\Psi_{x}\right)}_{m,p}=e^{-j4\pi \frac{R}{R_{0}c}\left(f_{m}+f_{c}\right)x_{p}}$ and ${\left(\Psi_{y}\right)}_{q,n}=e^{j4\pi \frac{R}{\lambda_{c}R_{0}}\theta_{n}y_{q}}$. N represents the noise and error matrix.

### 2.2. 2D Convolution Model for Turntable Radar

By applying the MF algorithm, the target reflectivity can be expressed as:

(11) $$\overset{⌢}{œ}\Psi_{x}^{H}Y\Psi_{y}^{H}.$$

Combining Formulas (10) and (11), we have:

(12) $$\overset{⌢}{œ}\Psi_{x}^{H}\Psi_{x}œ\Psi_{y}\Psi_{y}^{H}.$$

For every estimated scattering coefficient of target on the imaging area gird $\left(x_{p},y_{q}\right)$, we have:

(13) $$\overset{⌢}{\sigma }(p,q)=\sum_{p^{\prime }=1}^{P}\sum_{q^{\prime }=1}^{Q}\sigma (p^{\prime },q^{\prime })(\sum_{m=1}^{M}\sum_{n=1}^{N}e^{j4\pi \frac{R}{R_{0}c}\left(f_{m}+f_{c}\right)(x_{p}-{x^{\prime }}_{p})}e^{-j4\pi \frac{R}{\lambda_{c}R_{0}}\theta_{n}(y_{q}-{y^{\prime }}_{q})}).$$

Define the PSF as:

(14) $$h\left(x,y\right)=\sum_{m=1}^{M}\sum_{n=1}^{N}e^{j4\pi \frac{R}{R_{0}c}\left(f_{m}+f_{c}\right)x}e^{-j4\pi \frac{R}{\lambda_{c}R_{0}}\theta_{n}y}.$$

Then, we can rewrite (13) in 2D convolution form as:

(15) $$\overset{⌢}{\sigma }(p,q)\approx \sigma (p,q)*h(p,q)+n(p,q).$$

**Figure 2:**
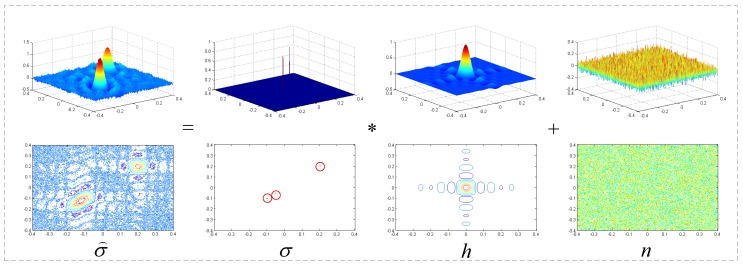
Schematic diagram of the convolution model.

Therefore, the signal after MF $\overset{⌢}{\sigma }(p,q)$ is a 2D convolution result of the target’s scattering coefficients and the PSF just as shown in Figure 2. For one target, the MF result will be proportional to the PSF of system, and for two targets that are closely spaced, the MF result will have only a single peak. Thus, the performance of the MF result is decided by the characteristic of PSF.

Then, the characteristic of the PSF will be analyzed. Computing the PSF according to (14) under a uniform sampling condition, we have:

(16) $$h\left(x,y\right)=e^{j2\pi \frac{R}{R_{0}}\left(\frac{\left(2f_{c}+M\Delta f\right)}{c}x-\frac{N\Delta \theta }{\lambda_{c}}y\right)}\frac{\sin\left(2\pi \frac{R}{R_{0}c}M\Delta fx\right)}{\sin\left(2\pi \frac{R}{R_{0}c}\Delta fx\right)}\frac{\sin\left(2\pi \frac{R}{R_{0}\lambda_{c}}N\Delta \theta y\right)}{\sin\left(2\pi \frac{R}{R_{0}\lambda_{c}}\Delta \theta y\right)}.$$

We can see from (16) that the PSF has a wide 2D main lobe decided by the bandwidth of the transmitted signal for the range direction and the total rotation angle for azimuth direction and a high sidelobe decided by the frequency sampling interval for the range direction and the angle sampling interval for the azimuth direction.

### 2.3. 2D Direct Deconvolution Problem from Undersampled Data

As mentioned above, the MF result can be treated as the 2D convolution of the target’s scattering coefficients and PSF, so the MF result is blurred by the PSF. For the purpose of getting the true target’s scattering coefficients, deconvolution is a simple solution.

In order to simply solve the deconvolution problem, we rewrite (15) in spatial spectrum domain form as follows:

(17) $${\overset{⌢}{\sigma }}_{F}(p,q)=\sigma_{F}(p,q)\cdot h_{F}(p,q)+n_{F}(p,q)$$

where ${\overset{⌢}{œ}}_{F}=F_{2}(\overset{⌢}{œ}),{œ}_{F}=F_{2}(œ)$, $h_{F}=F_{2}(h),n_{F}=F_{2}(n)$.

Therefore, the target’s scattering information could be restored by inverse filtering according to (17), which can be theoretically expressed as:

(18) $${\hat{\sigma }}_{F}\left(p,q\right)=\frac{{\overset{⌢}{\sigma }}_{F}\left(p,q\right)h_{F}^{*}\left(p,q\right)}{{\left|h_{F}(p,q)\right|}^{2}}=\sigma_{F}(p,q)+n_{F}(p,q)/h_{F}(p,q).$$

**Figure 3:**
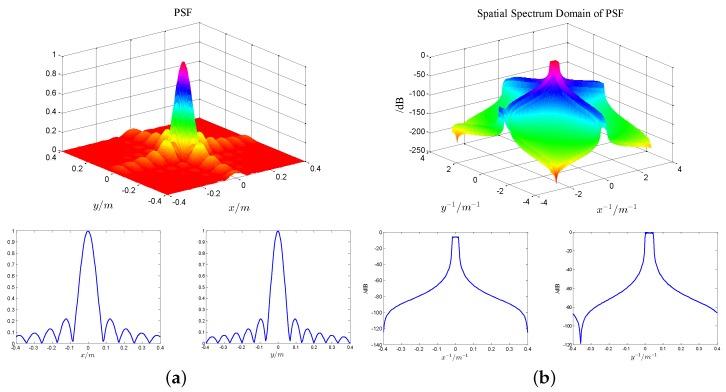
Point spread function (PSF) and its spatial spectrum domain with all data. (**a**) PSF and its two-dimensional profile; (**b**) spatial spectrum domain of PSF and its two-dimensional profile.

However, in practice, the result of (18) does not turn out so well because of the band limited characteristic of PSF, which can be clearly seen from Figure 3.

From (18) and Figure 3, we can see that the noise out of the band of PSF will result in tremendous amplification of noise and obtain valueless results since $1/h_{F}$ will be very large at those frequencies. Thus, the direct deconvolution processing is an ill-posed inverse problem.

**Figure 4:**
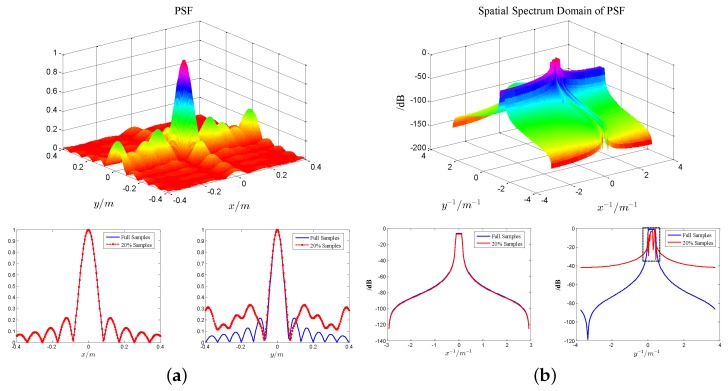
PSF and its spatial spectrum domain with 20% azimuth sampling data. (**a**) PSF and its 2D profile; (**b**) spatial spectrum domain of PSF and its 2D profile.

The performance of deconvolution is influenced by the bandpass characteristic of PSF, which is decided by the sampling of the frequency and rotation angle for turntable radar in the case of fixed bandwidth and rotation angle. In order to reduce the observation time and data transferred, we need to reduce the azimuth samples. However, this will result in performance deterioration of PSF in the band, and it is illustrated by Figure 4.

The undersampled result is shown in Figure 4, where only 20% azimuthal sampling data are available. Compared with the PSF and its spatial spectrum of full azimuth sampling data, we can find that the range dimension changes little because the range dimension sampling data have not been changed, but the azimuthal sidelobe of PSF is raised and leads to bad flatness in the band due to the reduction of azimuth data. Although the values of $h_{F}$ beyond the bandwidth increase, it is of little help to the performance improvement because the values are still too small. However, the deconvolution imaging performance will decline greatly when the values within the bandwidth reduce too much. Especially, when grating lobes of PSF appear, the deconvolution results would appear as a false target. Therefore, it is necessary to complete the undersampled data, and the recently proposed MC theory can be used for completion, which will be introduced in detail next.

## 3. Proposed 2D Deconvolution Algorithm with Proposed MC Algorithm

As noted before, the direct deconvolution is ill-posed for the situation of undersampled data. In this section, a 2D iterative deconvolution algorithm with MC is derived to solve the ill-posed problem, increase the operational accuracy and improve the resolution.

### 3.1. Reconstruction of Unknown Samples via MC

#### 3.1.1. MC Introduction

Before presenting our proposed MC algorithm, we introduce the problem of MC briefly. MC means recovering a low rank matrix based on partial knowledge of its entries, and it can be solved via solving a rank minimization problem [14]:

(19) $$\min_{X}\;\;\;rank\left(X\right)\;\;\;\;\;\;\;\;\;s.t.\;\;\;\;\;\;P_{\Omega }\left(X\right)=P_{\Omega }\left(M\right)$$

where M is the data matrix, which has some available sampled entries and $Y=P_{\Omega }\left(M\right)$ is defined as:

(20) $${\left[Y\right]}_{ij}=\left\{\begin{array}{c} {\left[M\right]}_{ij},\;\;\;\;\;\left(i,j\right)\in \Omega \\ 0,\;\;\;\;\;\;\;\;\;\;otherwise \end{array}\right.$$

where Ω is the set of indices of observed entries.

However, (19) is an NP-hard problem; the most commonly-used way is using a tightest convex relaxation optimization problem as follows:

(21) $$\min_{X}\;\;\;{∥X∥}_{*}\;\;\;\;\;\;\;\;\;s.t.\;\;\;\;\;\;P_{\Omega }\left(X\right)=P_{\Omega }\left(M\right).$$

It can be solved by the singular value thresholding (SVT) method [15]. It tends to underestimate the nonzero singular values; therefore, several recent studies have emphasized the benefit of nonconvex penalty functions compared to the nuclear norm for the estimation of singular values [19,20,21]. However, the nonconvex optimization problem suffers from numerous issues, such as spurious local minima and initialization issues. Taking into account the shortcomings of the traditional methods, we propose our MC method with a parameterized nonconvex penalty function.

#### 3.1.2. Proposed MC Algorithm by the Nonconvex Low Rank Minimization

**Figure 5:**
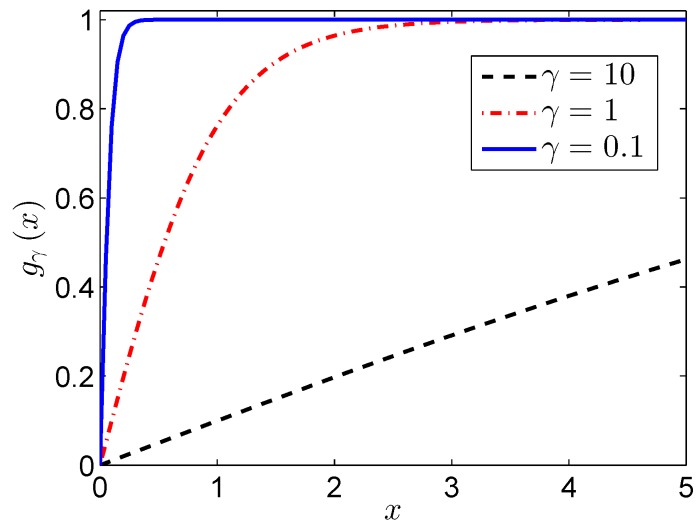
Illustration of the hyperbolic tangent function under different shape parameters.

Firstly, we introduce our parameterized nonconvex penalty function, which can be written as:

(22) $$g_{\gamma }\left(x\right)=\tanh\left(x/\gamma \right)=\frac{e^{x/\gamma }-e^{-x/\gamma }}{e^{x/\gamma }+e^{-x/\gamma }}$$

where *γ* is the shape parameter that determines the trend of its approximation to the rank function, just as shown in Figure 5, and the function with small *γ* is close to the rank function.

**Figure 6:**
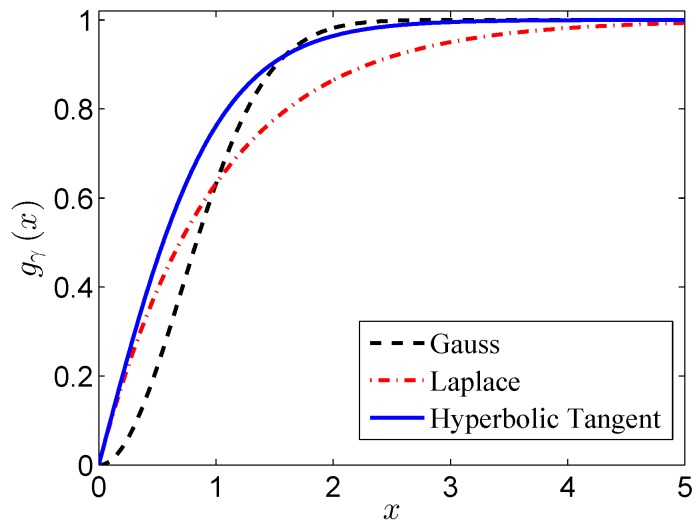
Illustration of different nonconvex penalty functions.

Compared to other commonly-used nonconvex penalty functions (Gaussian function, Laplace function, etc.), it has better approximation to the rank function under the same parameter (γ=1), which can be seen in Figure 6.

Then, we define our MC problem as:

(23) $$\min_{X}\;\;\;\sum_{i=1}^{r}g_{\gamma }\left(\sigma_{i}\left(X\right)\right)\;\;\;\;\;\;\;\;\;s.t.\;\;\;\;\;\;P_{\Omega }\left(X\right)=P_{\Omega }\left(M\right)$$

where $r=rank\left(X\right)$ and $\sigma_{i}\left(X\right)$ is the *i*-th singular value of matrix X. Obviously, the nonconvex constraint with a smaller shape parameter is more approximate to rank constraint. Formula (23) can also be expressed in another form:

(24) $$\min_{X}\;\;\;\sum_{i=1}^{r}g_{\gamma }\left(\sigma_{i}\left(X\right)\right)\;\;\;\;\;\;\;\;\;s.t.\;\;\;\;\;\;X+E=M,\;\;\;\;\;P_{\Omega }\left(E\right)=0.$$

Instead of using a hyperbolic tangent penalty function with a fixed shape parameter, parameter adjustment approximation is used to help in achieving the rank minimizer by gradually decreasing *γ*, which can avoid the above problems of spurious local minima and initialization issues.

Under a fixed parameter, we apply the augmented Lagrange multiplier method [22], which can guarantee quadratic convergence, and define the Lagrangian function as:

(25) LX,E,Z,μ=∑i=1rgγσiX+ReZ,M−X−E+μ2M−X−EF2

where Z is Lagrange multiplier matrix. μ>0 is the regularization parameter.

Then, the optimization problem (24) is equivalent to:

(26) $$\min_{X,\;\;P_{\Omega }\left(E\right)=0,Z,\mu }L\left(X,E,Z,\mu \right)$$

Using the alternating direction technique to solve the optimization problem (26):

(27) $$X^{k+1}=\arg\min_{X}L\left(X,E^{k},Z_{}^{k},\mu^{k}\right)$$

(28) $$E^{k+1}=\arg\min_{P_{\Omega }\left(E\right)=0}L\left(X^{k\;+\;1},E,Z_{}^{k},\mu^{k}\right)$$

(29) $$Z^{k+1}=Z^{k}+\mu^{k}\left(M-X^{k+1}-E^{k+1}\right)$$

(30) $$\mu^{k+1}=\rho \mu^{k},\;\;\;\;\;\;\;\;\;\;\;\;\;\;\;\;\;\;\;\;\rho >1.$$

It remains to compute the minimizer of (27) and (28), and note that for (27), it can be specifically rewritten as:

(31) $$X^{k+1}=\arg\min_{X}\sum_{i=1}^{r}g_{\gamma }\left(\sigma_{i}\left(X\right)\right)\;+\frac{\mu^{k}}{2}{∥M+\frac{Z^{k}}{\mu^{k}}-E^{k}-X∥}_{F}^{2}.$$

**Figure 7:**
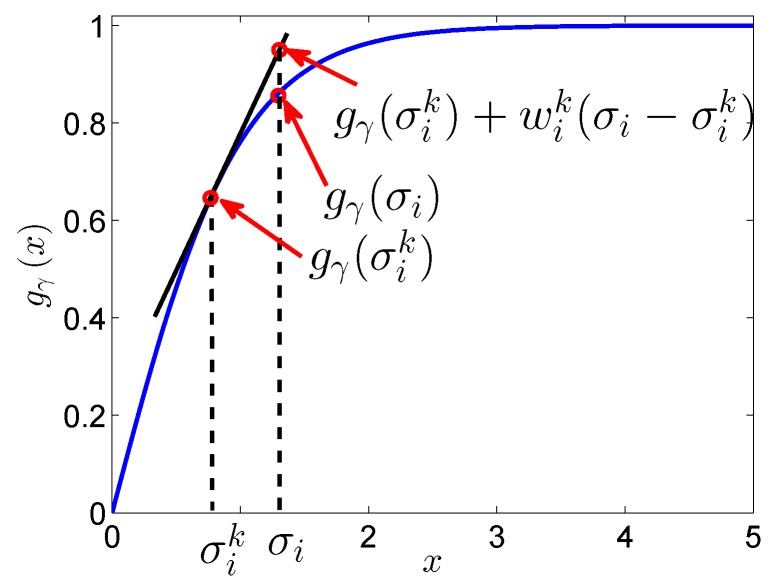
Gradient of the proposed nonconvex function.

Because the hyperbolic tangent function $g_{\gamma }$ is a concave function, according to Figure 7, we have:

(32) $$g_{\gamma }\left(\sigma_{i}\right)\leq g_{\gamma }\left(\sigma_{i}^{k}\right)+w_{i}^{k}\left(\sigma_{i}-\sigma_{i}^{k}\right)$$

where $w_{i}^{k}$ is the derivative of $g_{\gamma }$ on $\sigma_{i}^{k}$, calculated as:

(33) $$w_{i}^{k}=\partial g_{\gamma }\left(\sigma_{i}^{k}\right)=1/\gamma \cosh^{2}\left(\frac{\sigma_{i}^{k}}{\gamma }\right).$$

When the value of *γ* becomes large enough, the equality in (32) holds, which can be seen directly from Figure 5. Then, the proposed hyperbolic tangent function is the same as the nuclear norm.

Using majorization-minimization (MM) [23], we obtain $X^{k+1}$ by the following procedure:

(34) $$\begin{array}{cc} X^{k+1} & =\arg\min_{X}\sum_{i=1}^{r}\left(g_{\gamma }\left(\sigma_{i}^{k}\left(X\right)\right)+w_{i}^{k}\left(\sigma_{i}\left(X\right)-\sigma_{i}^{k}\left(X\right)\right)\right)\;+\frac{\mu^{k}}{2}{∥M+\frac{Z^{k}}{\mu^{k}}-E^{k}-X∥}_{F}^{2}\\ & =\arg\min_{X}\sum_{i=1}^{r}w_{i}^{k}\sigma_{i}\left(X\right)\;+\frac{\mu^{k}}{2}{∥M+\frac{Z^{k}}{\mu^{k}}-E^{k}-X∥}_{F}^{2}. \end{array}$$

**Figure 8:**
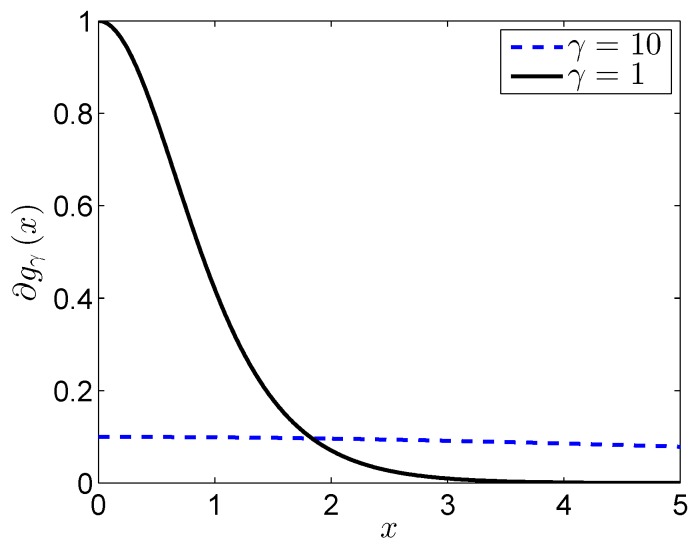
Gradient of $g_{\gamma }\left(x\right)$.

From (34), we can also give the reason that the nonconvex constraint is superior compared with the nuclear norm. For the nuclear norm, the punishment is the same for all of the variables, which is unfair to the large variables. However, for the nonconvex constraint, we can see that large variables have a small punishment according to Figure 8. If *γ* is large enough, the values of weighted coefficient w are almost exactly equal, which means our proposed nonconvex function is the same as the nuclear norm.

According to Lemma 1 [24], it has a closed form solution for (34) despite its nonconvexity.

Lemma 1: For any λ>0 and $0\leq w_{1}\leq \cdots \leq w_{r}$, the following problem (35) has a globally optimal solution:

(35) $$\min_{X}\lambda \sum_{i=1}^{r}w_{i}\sigma_{i}\left(X\right)\;+\frac{1}{2}{∥Y-X∥}_{F}^{2}$$

and it can be given by the weighted singular value thresholding as:

(36) $$\hat{X}\;=\;US_{\lambda w}\left(\Sigma \right)V^{T}$$

where $Y\;=\;U\Sigma V^{T}$ is the singular value decomposition (SVD) of Y and $S_{\lambda w}\left(\Sigma \right)$ is defined as:

(37) $$S_{\lambda w}\left(\Sigma \right)=diag\left\{{\left(\Sigma_{ii}-\lambda w_{i}\right)}_{+}\right\},\;\;\;\;\;\;\;\;{\left(\Sigma_{ii}-\lambda w_{i}\right)}_{+}=\left\{\begin{array}{c} \Sigma_{ii}-\lambda w_{i},\;\;\;\;\;\;\Sigma_{ii}-\lambda w_{i}>0\\ \;\;\;\;\;\;\;\;\;\;\;\;\;\;\;\;\;0,\;\;\;\;\;\;\;\;\;\;\;\;\;\;\;\;\;\;\;\;\;\Sigma_{ii}-\lambda w_{i}\leq 0 \end{array}\right..$$

Obviously, (34) has the same form as (35). Thus, we can solve (34) via Lemma 1, which can be given by:

(38) $$X^{k+1}\;=\;US_{w/\mu^{k}}\left(\Sigma \right)V^{T}$$

where $U\Sigma V^{T}\;=\;svd\left(M+Z^{k}/\mu^{k}-E^{k}\right)$.

For (28), it can be rewritten as:

(39) $$E^{k+1}=\arg\min_{P_{\Omega }\left(E\right)=0}\frac{\mu^{k}}{2}{∥M-X^{k+1}+\frac{Z^{k}}{\mu^{k}}-E∥}_{F}^{2}.$$

Thus, we can solve the problem (39) to update $E^{k+1}$ by:

(40) $$E^{k+1}=P_{\bar{\Omega }}\left(M-X^{k+1}+\frac{Z^{k}}{\mu^{k}}\right)$$

where $\bar{\Omega }$ is the complement set of Ω.

Then, the procedure of the proposed MC algorithm is described in Algorithm 1. The out loop is used for updating shape parameter *γ*.

**Algorithm 1** Procedure of the proposed matrix completion (MC) algorithm
  1:**Initialization:** $X_{0}=M,\;Z^{0}=0,\;E^{0}=0\;,\mu^{0}=1/{∥X_{0}∥}_{2},\;\gamma_{0}>0,\;\beta <1$  2:**For** l=0,⋯,L  3:        $X^{0}=X_{l}$  4:        **For** k=1,⋯,K  5:               Update $X^{k}$ via (38);  6:               Update $E^{k}$ via (40);  7:               Update $Z^{k}$ via (29);  8:               Update $\mu^{k}$ via (30);  9:               if ${{∥X^{k+1}-X^{k}∥}_{F}/∥X^{k}∥}_{F}<\varepsilon$, then break;10:        **End**11:        $X_{l+1}=X^{k+1},\;\;\;\;\gamma_{l+1}=\beta \gamma_{l}$12:        if ${{∥X_{l+1}-X_{l}∥}_{F}/∥X_{l}∥}_{F}<\varepsilon$, then break;13:**End**14:**Output:** $X_{l+1}$


#### 3.1.3. Low Rank Echo Matrix Model for Turntable Radar

**Figure 9:**
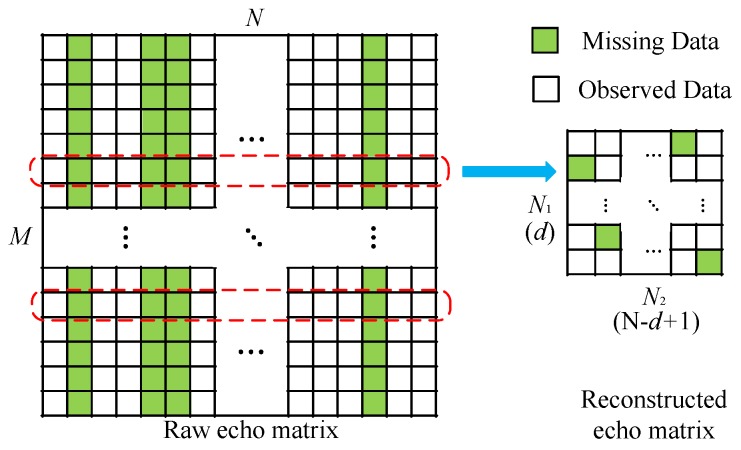
Matrix reconstruction for undersampled azimuth data.

In this paper, we are more interested in reducing the number of azimuthal samples. However, it is difficult to directly use the proposed MC algorithm to complete the missing elements in this case because the echo matrix to be restored does not satisfy the strong incoherence property (SIP) [14]. Therefore, we rearrange the original echo matrix and construct a new low rank echo matrix that satisfies SIP. The work in [17] proposed a method for matrix construction, which constructed a small matrix with a size of $N_{1}\times N_{2}=N$ for each echo vector of the same frequency. Additionally, [18] introduced another way to reshape the stepped frequency echo into a Hankel matrix form of size $d\times \left(N-d+1\right)$, which is much larger than $N_{1}\times N_{2}$. Therefore, the reconstructed matrix has a better low rank property. However, the exiting forms for matrix reconstruction should process every frequency echo separately just as shown in Figure 9; it is complex, and the low rank property is also not very good because of limited samples. In this paper, we propose a new form of matrix reconstruction using all of the echo data together, which can improve the low rank property of the echo matrix and reduce the computational complexity.

According to (10), under the assumption that the target satisfies the a priori point scattering, the echo of the *m*-th frequency can be expressed as:

(41) $$Y\left(m,n\right)=\sum_{k=1}^{K}e^{-j4\pi \frac{R}{R_{0}c}\left(f_{m}+f_{c}\right)x_{k}}\sigma \left(x_{k},y_{k}\right)e^{j4\pi \frac{R}{\lambda_{c}R_{0}}\theta_{n}y_{k}}$$

where *K* is the number of scattering points.

Constructing a small matrix $Y_{m}$ as (42) using the *m*-th row elements of original matrix Y:

(42) $$Y_{m}={\left[\begin{array}{cccc} Y\left(m,1\right) & Y\left(m,N_{1}+1\right) & \cdots & Y\left(m,\left(N_{2}-1\right)N_{1}+1\right)\\ Y\left(m,2\right) & Y\left(m,N_{1}+2\right) & \cdots & Y\left(m,\left(N_{2}-1\right)N_{1}+2\right)\\ \vdots & \vdots & \ddots & \vdots \\ Y\left(m,N_{1}\right) & Y\left(m,2N_{1}\right) & \cdots & Y\left(m,N_{2}N_{1}\right) \end{array}\right]}_{N_{1}\times N_{2}}.$$

Additionally, it can be written as:

(43) $$Y_{m}=A_{m}œB$$

where $œ=diag\left(\sigma \left(x_{1},y_{1}\right),\cdots ,\sigma \left(x_{K},y_{K}\right)\right)$ and matrices $A_{m}$, B are defined as:

(44) $$A_{m}\;=\;\;{\left[\begin{array}{cccc} a_{1}^{}\left(m,1\right) & a_{2}^{}\left(m,1\right) & \cdots & a_{K}^{}\left(m,1\right)\\ a_{1}^{}\left(m,2\right) & a_{2}^{}\left(m,2\right) & \cdots & a_{K}^{}\left(m,2\right)\\ \vdots & \vdots & \ddots & \vdots \\ a_{1}^{}\left(m,N_{1}\right) & a_{2}^{}\left(m,N_{1}\right) & \cdots & a_{K}^{}\left(m,N_{1}\right) \end{array}\right]}_{N_{1}\times K}\;\;\;\;\;\;\;\;\;\;\;\;,B\;=\;\;{\left[\begin{array}{cccc} b_{1}^{}\left(1\right) & b_{1}^{}\left(2\right) & \cdots & b_{1}^{}\left(N_{2}\right)\\ b_{2}^{}\left(1\right) & b_{2}^{}\left(2\right) & \cdots & b_{2}^{}\left(N_{2}\right)\\ \vdots & \vdots & \ddots & \vdots \\ b_{K}^{}\left(1\right) & b_{K}^{}\left(2\right) & \cdots & b_{K}^{}\left(N_{2}\right) \end{array}\right]}_{K\times N_{2}}$$

where,

(45) $$a_{k}\left(m,n\right)=e^{-j4\pi \frac{R}{R_{0}c}\left(f_{m}+f_{c}\right)x_{k}}e^{j4\pi \frac{R}{\lambda_{c}R_{0}}n\Delta \theta y_{k}},b_{k}\left(n\right)=e^{j4\pi \frac{R}{\lambda_{c}R_{0}}\left(n-1\right)N_{1}\Delta \theta y_{k}}$$

Then, we can reconstruct matrix Y as:

(46) $$\begin{array}{c} Y_{new}={\left[\begin{array}{cccc} Y_{1} & Y_{M_{1}+1} & \cdots & Y_{\left(M_{2}-1\right)M_{1}+1}\\ Y_{2} & Y_{M_{1}+2} & \cdots & Y_{\left(M_{2}-1\right)M_{1}+2}\\ \vdots & \vdots & \ddots & \vdots \\ Y_{M_{1}} & Y_{2M_{1}} & \cdots & Y_{M_{2}M_{1}} \end{array}\right]}_{M_{1}N_{1}\times M_{2}N_{2}}\\ ={\left[\begin{array}{c} A_{1}C^{0}\\ A_{1}C^{1}\\ \vdots \\ A_{1}C^{M_{1}-1} \end{array}\right]}_{M_{1}N_{1}\times K}\cdot {\left[\begin{array}{cccc} C^{0}œB & C^{M_{1}}œB & \cdots & C^{\left(M_{2}-1\right)M_{1}}œB \end{array}\right]}_{K\times M_{2}N_{2}} \end{array}$$

where $M=M_{1}\times M_{2}$ and:

(47) $$C=diag\left(c_{1},c_{2},\cdots ,c_{K}\right),\;\;\;\;\;\;\;c_{k}=e^{-j4\pi \frac{R}{R_{0}c}\Delta fx_{k}}.$$

Obviously, the new echo matrix $Y_{new}$ is low rank with $rank\left(Y_{new}\right)\leq K\ll \min\left\{M_{1}N_{1},M_{2}N_{2}\right\}$. For the new matrix $Y_{new}$, its missing elements are randomly distributed, so it satisfies the SIP. Besides, it has a larger size than [17,18] with the same rank *K*, which means that it has better low rank property. Additionally, it only needs one matrix reconstruction for all of the elements, which is much less than the methods of [17,18]. Therefore, we can use the proposed MC algorithm for the new echo matrix. After MC, we reshape $Y_{new}$ to the original echo matrix Y.

### 3.2. 2D Proposed Deconvolution Algorithm for High Resolution Radar Imaging

We have mentioned in Section 2.3 that direct deconvolution suffers from the ill-posed problem. In this part, we will introduce a new deconvolution method to reduce the ill-posed condition and achieve high resolution radar imaging.

For Formula (15), it can be expressed in matrix vector form as:

(48) g=Hu+n

where $g=vec(\overset{⌢}{œ})$, $u=vec\left(œ\right)$ and H is a block circulant matrix formed by the PSF.

Additionally, for the direct deconvolution algorithm, it can be written in vector form as:

(49) $$u={\left(H^{H}H\right)}^{-1}H^{H}g.$$

Regarding the ill-posed problem for direct deconvolution, a regularization method is given by the $l_{2}$ norm as:

(50) $$\min_{u}J\left(u\right)=\frac{1}{2}{∥g-\mathrm{Hu}∥}_{2}^{2}+\lambda {∥u∥}_{2}^{2}.$$

The optimization result of (50) can be written as:

(51) $$u={\left(H^{H}H+\lambda I\right)}^{-1}H^{H}g$$

which can also be expressed in frequency domain form as Wiener filtering:

(52) $${\hat{\sigma }}_{F}\left(p,q\right)=\frac{h_{F}^{*}\left(p,q\right){\overset{⌢}{\sigma }}_{F}\left(p,q\right)}{{\left|h_{F}(p,q)\right|}^{2}+\lambda }.$$

The regularization parameter *λ* is difficult to choose and usually determined according to experience. In order to avoid the problem of parameter selection, we modify the the Wiener filter algorithm to further improve the performance of deconvolution. Here, we also use the $l_{2}$ norm function as the regularization constraint and define the corresponding augmented Lagrangian function as:

(53) Lu,y,μ=μ2g−Hu22+Rey,g−Hu+u22.

Then, the ALMupdate takes the form of:

(54) $$u^{k+1}=\arg\min_{u}L\left(u,y^{k},\mu^{k}\right)$$

(55) $$y^{k+1}=y^{k}+\mu^{k}\left(g-Hu^{k+1}\right)$$

(56) $$\mu^{k+1}=\rho u^{k},\;\;\;\;\;\;\;\;\rho >1.$$

We can solve the optimization problem (54) as:

(57) $$u^{k+1}=\arg\min_{u}\frac{1}{2}{∥g+\frac{y^{k}}{\mu^{k}}-\mathrm{Hu}∥}_{2}^{2}+\frac{1}{\mu^{k}}{∥u∥}_{2}^{2}.$$

Additionally, it can be solved with:

(58) $$u^{k+1}={\left(H^{H}H+\frac{1}{\mu^{k}}I\right)}^{-1}H^{H}\left(g+\frac{y^{k}}{\mu^{k}}\right).$$

In order to reduce the computational load, we rewrite the iterative process in the frequency domain. Let $y_{F}=F_{2}\left\{reshape\left(y,P,Q\right)\right\}$; the frequency domain form of (55) and (58) can be expressed as:

(59) $$y_{F}^{}{\left(p,q\right)}^{k+1}=y_{F}^{}{\left(p,q\right)}^{k}+\mu^{k}\left({\overset{⌢}{\sigma }}_{F}\left(p,q\right)-h_{F}\left(p,q\right)\sigma_{F}{\left(p,q\right)}^{k+1}\right)$$

(60) $$\sigma_{F}{\left(p,q\right)}^{k+1}=\frac{h_{F}^{*}\left(p,q\right)\left({\overset{⌢}{\sigma }}_{F}\left(p,q\right)+\frac{y_{F}{\left(p,q\right)}^{k}}{\mu^{k}}\right)}{{\left|h_{F}\left(p,q\right)\right|}^{2}+\frac{1}{\mu^{k}}}.$$

Further to improve the performance, we combine the soft thresholding function [25] to reduce the sidelobe caused by noise and model errors, which can be written as:

(61) $$\sigma_{F}{\left(p,q\right)}^{k+1}=F_{2}\left(soft\left(F_{2}^{-1}\left\{\frac{h_{F}^{*}\left(p,q\right)\left({\overset{⌢}{\sigma }}_{F}\left(p,q\right)+\frac{y_{F}{\left(p,q\right)}^{k}}{\mu^{k}}\right)}{{\left|h_{F}^{}\left(p,q\right)\right|}^{2}+\frac{1}{\mu^{k}}}\right\},\tau \right)\right)$$

where *τ* is decided by the noise level and:

(62) $$\hat{x}=soft\left(y,\tau \right)≐\frac{\max\left\{\left|y\right|-\tau ,0\right\}}{\max\left\{\left|y\right|-\tau ,0\right\}+\tau }y.$$

Then, we can get the proposed deconvolution algorithm as shown in Algorithm 2.

**Algorithm 2** Proposed 2D Deconvolution Algorithm
  1:**Initialization:** $y_{F}^{0}=0,\;\;\;\;\mu^{0}=1/{∥F_{2}^{-1}\left\{{\overset{⌢}{œ}}_{F}\right\}∥}_{F},\;\;\tau >0$  2:**For** k=1,⋯,K  3:        Update ${œ}_{F}^{k}$ via (61);  4:        Update $y_{F}^{k}$ via (59);  5:        Update $\mu^{k}$ via (56);  6:        if ${{∥{œ}_{F}^{k+1}-{œ}_{F}^{k}∥}_{F}/∥{œ}_{F}^{k}∥}_{F}<\varepsilon$, then break;  7:**End**  8:**Output:** $F_{2}^{-1}\left\{{œ}_{F}^{k+1}\right\}$


At the beginning of the iteration, 1/μ is large, which means that the main lobe of the recovery result is wide, but the sidelobe caused by the noise is very low. With the increase of iterations, 1/μ becomes smaller, and the sidelobe caused by noise raises, but the denoise operation can remove the noise effectively and improve the performance of recovery result.

**Figure 10:**
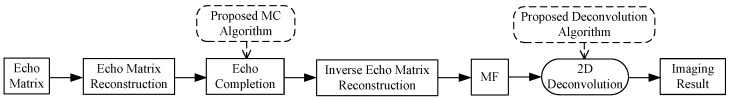
Diagram of the proposed method.

By combining the proposed MC algorithm with the proposed matrix reconstruction method and the proposed 2D deconvolution algorithm, our proposed method for radar imaging under the situation of azimuth undersampled data can be described as shown in Figure 10.

## 4. Simulation and Experimental Results

In this section, we present several numerical simulation and experimental results to illustrate the performance of the proposed method. All of the results are performed by using MATLAB R2014a on a PC equipped with an Inter Core i5-4590 CPU, 3.3 GHz and 12 GB memory. The normalized mean square error (NMSE) is used for evaluating the performance of simulation results. The image entropy (IE) [26] and image contrast (IC) [27] are used for measuring the performance of experimental results, where low values of IE and high values of IC generally mean that the image is well recovered. The most commonly-used classical MC algorithms, such as SVT [15], the inexact ALM method [28], etc., and deconvolution algorithms, like Wiener filtering algorithm [29], iterative constraint deconvolution (CID) [30], etc., are selected for comparison.

### 4.1. Numerical Simulations

The simulation conditions are given in Table 1. Ten point targets are randomly distributed in the imaging area. We set $M_{1}=10,\;M_{2}=20,\;N_{1}=20,\;N_{2}=10$ for the echo reconstruction.

**Figure 11:**
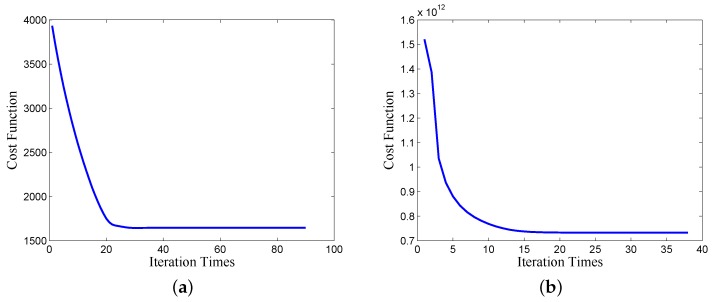
Convergence curves of (**a**) the proposed MC algorithm and (**b**) the proposed 2D deconvolution algorithm.

Firstly, during the iterative process, the cost functions for the proposed MC and deconvolution algorithm keep decreasing after each iteration, as shown in Figure 11, which further demonstrates the convergence of the proposed algorithms.

**Figure 12:**
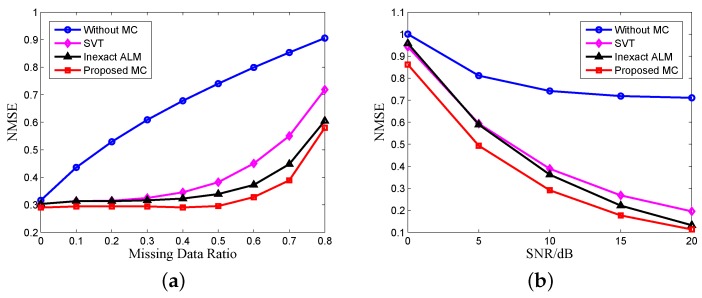
MC recovery comparison of (**a**) the missing data ratio (SNR = 10 dB) and (**b**) SNR (missing data ratio = 0.5).

**Figure 13:**
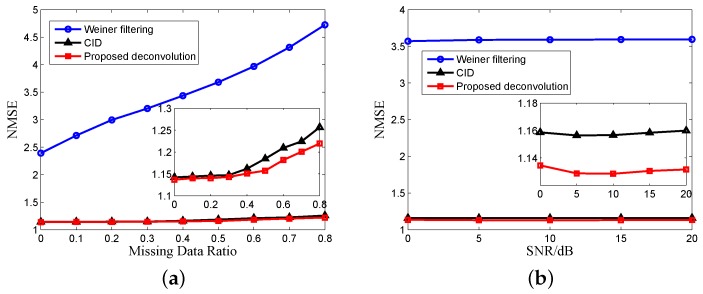
Deconvolution comparison of (**a**) the missing data ratio (SNR = 10 dB) and (**b**) SNR (missing data ratio = 0.5).

For the next simulation, we use the matrix reconstruction method of [17] + SVT and the matrix reconstruction method of [18] + the inexact ALM method for comparing the MC algorithms. The winnerfiltering algorithm and CID are used for comparing deconvolution algorithms. Figure 12 shows the NMSE of results without and with MC versus the number of missing data and echo SNR using different MC algorithms, which is averaged over 100 Monte Carlo trials. The missing data are randomly distributed. We can see that our proposed MC algorithm has the best recovery result because our reconstructed matrix has the best low rank property, and our proposed MC algorithm with the nonconvex constraint has better performance than the traditional nuclear norm. SNR has greater impact on the MC algorithm because when the SNR is very low, the low rank property of the echo matrix will decrease rapidly. Figure 13 is the NMSE of the results versus the number of missing data and SNR using different deconvolution algorithms. It can be seen from Figure 13a that the performance of the deconvolution algorithms becomes worse with the increase of missing data. The conclusion is similar to our previous analysis and illustrates the necessity of echo completion. In Figure 13b, the SNR has little influence on the performance of deconvolution because after MF, the noise is suppressed, and the SNR of echo is enhanced.

**Figure 14:**
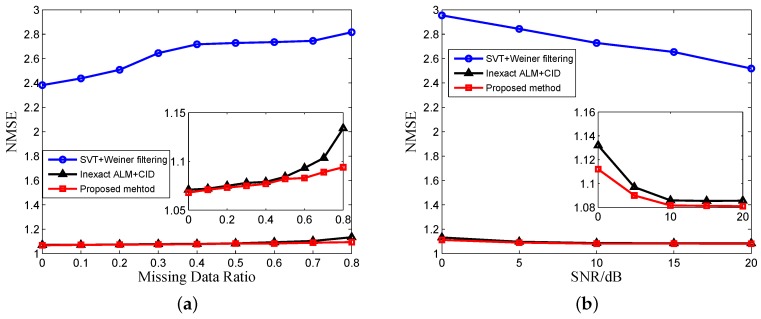
Imaging results comparison of (**a**) the missing data ratio (SNR = 10 dB) and (**b**) SNR (missing data ratio = 0.5).

The NMSE results after both MC and deconvolution versus the number of missing data and SNR are shown in Figure 14. Comparing Figure 13a with Figure 14a, we can see that the MC can improve the performance of deconvolution with undersampled data. The recovery results in Figure 14b are affected by SNR, which is different from Figure 13b, because the performance of MC algorithms is affected by noise, as shown in Figure 12b. In practical applications, the echo SNR after MF is usually not very high, and our proposed method will have better performance. From Figure 12, Figure 13 and Figure 14, we can see the superiority of the proposed algorithm compared to traditional algorithms, and we choose the matrix reconstruction method of [18] + the inexact ALM method + CID for comparison in the following processing of experimental data.

### 4.2. Experimental Results

In this subsection, some experimental results are reported to illustrate the validity and effectiveness of the proposed method. We also show the reconstructed results by MF and the matrix reconstruction method of [18] + the inexact ALM method + the CID method for comparison.

#### 4.2.1. ISAR Imaging

As we all know, after translational motion compensation, the ISAR imaging is similar to the turntable model in this paper under the assumption that $D=0,\;Z_{0}=0$. In this subsection, the quasi real data of an airplane (MIG25) provided by the U.S. Naval Research Laboratory are used, which transmit the stepped frequency (SF) signal with the center frequency of 9 GHz and the bandwidth of 512 MHz and consist of 512 cross-range samplings with 128 samplings used here and 64 range samplings.

**Figure 15:**
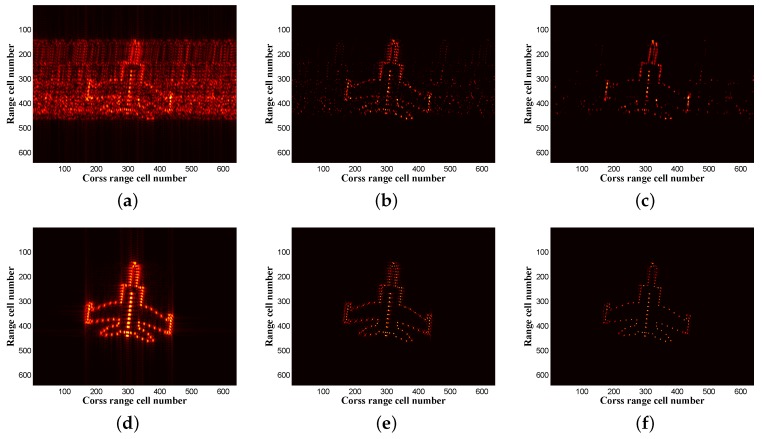
Imaging results of the MIG25. (**a**) Matched filter (MF) result without MC; (**b**) iterative constraint deconvolution (CID) result without MC; (**c**) proposed deconvolution algorithm result without MC; (**d**) MF result with all data; (**e**) CID result with the inexact ALMmethod; (**f**) proposed deconvolution algorithm result with the proposed MC algorithm.

To illustrate the validity of the proposed method, the reconstructed results of different methods with 25% cross-range data (32 samplings) are compared in Figure 15, where we set $M_{1}=8$, $M_{2}=8$, $N_{1}=16$, $N_{2}=8$. The observed data are randomly selected. Figure 15a–c shows the recovery results by different methods without MC. Figure 15d–f is the recovery results by different methods with MC. We can clearly see that without MC, the results of MF and deconvolution appear as many false scattering points, which are caused by partial data missing, and the false points can be eliminated through MC shown as Figure 15d–f. Our proposed method has better recovery performance with a clear object image by comparing Figure 15e with Figure 15f, which can be further validated by the IE and IC values of the recovered ISAR images under different methods calculated in Table 2. Although only 25% of the data are used here and the target model consists of many scattering points, good imaging results are obtained due to the high SNR of echo. Both the IC and the IE values confirm image quality improvement when the proposed method is used.

#### 4.2.2. Turntable Radar Imaging

**Figure 16:**
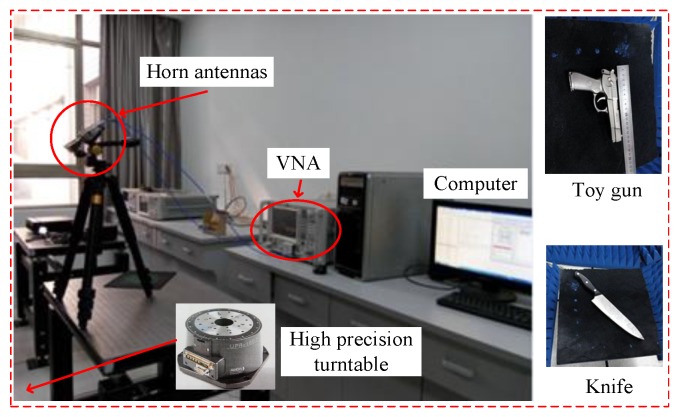
Surveillance scene of the experiment.

An SF radar is used in the experiment as shown in Figure 16, which contains a vector network analyzer (VNA) operating within 0.1∼40 GHz, two horn antennas, a high precision turntable and a control computer. The targets are four metal balls with an 8-mm diameter, a toy gun and a knife. The measurement parameters are shown in Table 3. We divide the azimuthal data into 72 segments, and for each segment, we can use the proposed model for approximation. Then, we process every segment and fuse the results of all of the segments in order to avoid the changing of the scattering characteristic due to different observation angles in the case of a large rotation angle. Fifty percent of cross-range data chosen randomly are used here. We set $M_{1}=16,\;M_{2}=16,\;N_{1}=4,\;N_{2}=5$ for matrix reconstruction.

**Figure 17:**
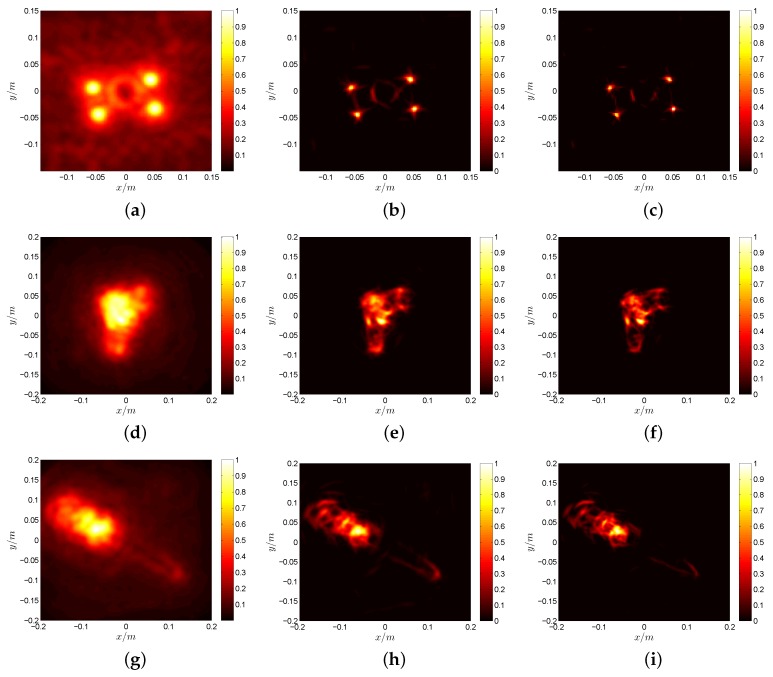
Imaging results of metal balls: (**a**) MF result with all data; (**b**) CID result with the inexact ALM method; (**c**) proposed deconvolution algorithm result with the proposed MC algorithm. Imaging results of the toy gun: (**d**) MF result with all data; (**e**) CID result with the inexact ALM method; (**f**) proposed deconvolution algorithm result with the proposed MC algorithm. Imaging results of the knife: (**g**) MF result with all data; (**h**) CID result with the inexact ALM method; (**i**) proposed deconvolution algorithm result with the proposed MC algorithm.

Figure 17 shows the results of MF, the inexact ALM method + the CID method and the proposed method, respectively, by using 50% of the azimuth full data. The used data are randomly selected. In this experiment, we use three different kinds of targets to test our proposed method, including the simple target of metal balls and complex targets, like toy gun and knife. The metal balls can be treated as the scattering model with fewer targets, while the toy gun and knife are composed of a large number of scattering points. Therefore, the imaging results of toy gun and knife are not as good as metal balls due to the poor low rank property under a large number of scattering points. It should be noticed that the handle of the knife has diffuse reflectivity, and the blade has specular reflectivity, so the strong scattering points of the knife are located in the handle, and the blade is not very clear, as shown in Figure 17g–i. Figure 17a,d,g is the results of MF, and they are blurred by the convolution effect under limited bandwidth and rotation angle. After the process of MC and deconvolution, the recovered images become much clearer and more conducive to target recognition. Our proposed method has a narrower main lobe and can obtain the object contour clearly compared to traditional methods. Additionally, the image quality improvement of our proposed method can be further confirmed according to the IE and IC values shown in Table 4. Our proposed method does not get a very significant performance boost for the complex targets according to Figure 17 and Table 4. This is mainly due to two following reasons. The low rank property of the reconstructed echo matrix is not very good because of the large number of scattering points and the small size of the matrix. The SNR of the raw data is not high under the constraint of transmit power and the influence of system noise and errors.

### 4.3. Discussion

Based on the above content, our proposed method can be mainly divided into two parts: MC and deconvolution. For the algorithm of MC, the number of observed data (*m*), the size of the matrix ($n=\max(M_{1}N_{1},M_{2}N_{2})$), the rank of the matrix (*r*) and SNR are the main factors that affect the performance of MC. According to the conclusion of Candès et al. [13,14], $m\geq C\mu^{2}nr\log^{6}n$ should be satisfied, where *C* is a numerical constant and *μ* is the strong incoherence parameter. This means that a fixed matrix with a larger rank demands more observed data. The result of MC $\hat{X}$ obeys ${∥X-\hat{X}∥}_{F}\leq 4\sqrt{\frac{\left(p+2\right)\min\left(M_{1}N_{1},M_{2}N_{2}\right)}{p}}\delta +2\delta$, where p=m/MN, *δ* is decided by the noise level and satisfies $\delta^{2}\leq \left(m+\sqrt{8m}\right)\sigma^{2}$ with high probability, and *σ* is the standard deviation of the white noise. Obviously, less missing data and a high SNR lead to high recovery accuracy of MC, which can also be obtained from our simulations and experimental results. From Figure 12, we can see that when the missing data ratio is smaller than 0.5 and the SNR is larger than 10 dB, the recovery result of MC shows good performance. For the deconvolution, its performance is affected by SNR and the spectral characteristics of PSF. It is worth noting that MF can improve the SNR of echo because MF is based on the maximum SNR criterion. Additionally, after MF, the SNR of the signal satisfies the requirement of the deconvolution algorithms, and the increased SNR of raw data has little impact on the performance promotion of the deconvolution results, which can be seen from Figure 13b. The PSFs under different missing data ratios are different, and the deconvolution has better performance when the ratio is smaller than 0.5, as shown in Figure 13a. Therefore, if the missing data ratio is smaller than 0.5 and SNR is larger than 10 dB, our proposed method has a significant performance boost. The requirement of observed data will reduce if the SNR increases, and the requirement of SNR will decrease if the number of observed data increases. In this paper, the influence of the number of scattering points on the performance of the proposed method is not discussed under the assumption that the strong scattering points for the turntable radar are sparsely distributed under high frequency. For the target with too many scattering points, the performance of the proposed method will decrease because it does not meet the condition of MC. In this situation, more observed data are needed to improve the low rank property of the echo matrix.

## 5. Conclusions

A novel method for high resolution turntable radar imaging with undersampled data of the cross-range is presented in this paper. The main work and contributions are summarized as follows:

1. Based on the result of MF, the 2D convolution model for turntable radar with arbitrary signals was constructed. Considering the blurring of the image caused by convolution, a novel 2D iterative deconvolution algorithm had been proposed for removing the convolution effect and achieving a high resolution radar imaging result. Through the analysis of the simulation and experimental results, the performance of the deconvolution algorithm could be further improved from the following two aspects: optimization of PSF and improvement of SNR.
2. In order to compress the observed data for reducing the number of transmit-receive channels and the difficulties of data transmission and storage, the MC algorithm with the nonconvex constraint and a novel echo matrix reconstruction method were proposed to complete the cross-range missing data for improving the characteristic of PSF. The data compression ratio was decided by the low rank property and SNR of the echo matrix.
3. Extensive simulations and experiments with simple and complex targets had been conducted to validate and compare the performance of the proposed method with several popular solvers.

According to the analysis of our proposed method in this paper, in the future, we will further improve our method from the following aspects:

1. In each iteration of the proposed MC algorithm, the most expensive operation is SVD. The random projection method [31] can be used to reduce the computation load of SVD, which operates SVD with a matrix of a much smaller size.
2. The noise of the system can be further suppressed by low rank matrix denoising technology in the image processing field using the low rank property of echo before and after MF.
3. Taking the fact that there are external disturbances and model mismatch into account, the error tolerance of the proposed method should be considered.
4. A larger size of the matrix should be reconstructed to improve the low rank property for a complex target with many scatterers.
5. The characteristic of the system PSF can be improved to enhance the imaging performance by extending our proposed method to other imaging systems, such as the metamaterial imaging system [32,33] and the coincidence imaging radar system [34], both of which have a better PSF than the traditional radar system under fixed bandwidth and synthetic aperture.

## Figures and Tables

**Table 1:** Simulation parameters.

Parameter	Value
Distance between the transmitting and receiving antenna *D*	0.04 m
Distance from antenna to YZ plane *R*	1 m
Distance from antenna to XY plane $Z_{0}$	0.7 m
Frequency step ∆f	20 MHz
Number of frequency *M*	200
Rotation angle step ∆θ	$0.025^{\circ }$
Number of rotation angle *N*	200
Wavelength $\lambda_{c}$	0.01 m
Imaging scene	1 m ×1 m
Number of grids for range direction *P*	100
Number of grids for azimuth direction *Q*	100

**Table 2:** Values of image entropy (IE) and image contrast (IC) in different schemes.

	(a)	(b)	(c)	(d)	(e)	(f)
**IE**	11.5975	8.7454	8.1093	10.0024	8.0969	7.3564
**IC**	2.4010	10.5054	14.1812	5.6070	13.7945	19.4382

**Table 3:** Experimental parameters.

Parameter	Value
Distance between the transmitting and receiving antenna *D*	0.04 m
Distance from antenna to YZ plane *R*	1.25 m
Distance from antenna to XY plane $Z_{0}$	0.6 m
Frequency step ∆f	40 MHz
Number of frequency *M*	256
Rotation angle step ∆θ	$0.25^{\circ }$
Number of rotation angle *N*	1440
Wavelength $\lambda_{c}$	0.01 m
Number of grids for range direction *P*	200
Number of grids for azimuth direction *Q*	200

**Table 4:** Values of IE and IC in different schemes.

Target	Parameter	MF	Inexact ALM method + CID	Proposed method
**metal balls**	**IE**	9.3976	5.1976	4.4626
	**IC**	2.6098	20.2438	29.0054
**toy gun**	**IE**	8.4137	7.0569	6.6240
	**IC**	3.7964	6.9976	9.0716
**knife**	**IE**	8.7608	7.0287	6.5888
	**IC**	3.4945	7.7789	9.9342
